# DHA Ameliorates Cognitive Ability, Reduces Amyloid Deposition, and Nerve Fiber Production in Alzheimer’s Disease

**DOI:** 10.3389/fnut.2022.852433

**Published:** 2022-06-15

**Authors:** Min Xiao, Wei Xiang, Yashu Chen, Nan Peng, Xiubo Du, Shuhuan Lu, Yao Zuo, Boling Li, Yonggang Hu, Xiangyu Li

**Affiliations:** ^1^College of Life Science and Technology, Huazhong Agricultural University, Wuhan, China; ^2^CABIO Biotech (Wuhan) Co., Ltd., Wuhan, China; ^3^Key Laboratory of Oil Crop Biology and Genetic Breeding, Oil Crops Research Institute, Ministry of Agriculture, Chinese Academy of Agricultural Sciences, Wuhan, China; ^4^Shenzhen Key Laboratory of Microbial Genetic Engineering, College of Life Sciences and Oceanography, Shenzhen University, Shenzhen, China

**Keywords:** Alzheimer’s disease, docosahexaenoic acid, omega-3, PUFAs, neurodegenerative disease, Aβ, NFT, liquid NMR

## Abstract

**Background:**

The etiology of Alzheimer’s disease (AD) is very complex. Docosahexaenoic acid (DHA) is important in cognitive ability and nervous system development. A limited number of studies have evaluated the efficacy of DHA in the treatment of AD.

**Introduction:**

We detected neurofibrillary tangles (NFT) in the hippocampus and cortex of transgenic mice brain through silver glycine staining. We determined the activity of neurons by staining Nissl bodies, used liquid NMR to detect metabolites in the brain, and functional magnetic resonance imaging results to observe the connection signal value between brain regions.

**Materials and Methods:**

We fed 3-month-old APP/PS1 double transgenic mice with DHA mixed feeds for 4 months to assess the effects of DHA on cognitive ability in AD mice through the Morris water maze and open field tests. To evaluate its effects with AD pathology, continuous feeding was done until the mice reached 9 months of age.

**Results:**

Compared to AD mice, escape latency significantly decreased on the fifth day while swimming speed, target quadrant stay time, and the crossing number of platforms increased by varying degrees after DHA treatment. Brain tissue section staining revealed that DHA significantly reduced Aβ and nerve fibers in the brain of AD mice.

**Conclusion:**

DHA significantly reduced the deposition of Aβ in the brain and inhibited the production of nerve fibers, thereby increasing cognitive abilities in AD mice. In addition, DHA suppressed blood lipid levels, and restored uric acid and urea levels, implying that DHA is a potential therapeutic option for early AD.

## Introduction

Alzheimer’s disease (AD) is a progressive neurodegenerative disease that is characterized by cognitive difficulties, reduced language abilities, weakened learning and memory abilities, and motor dysfunctions (1). Not only does it cause great harm to the patients, it is also associated with a heavy socio-economic burden on the family and society. Currently, senile plaques (SP) formed by Amyloid β-peptide (Aβ) deposition and nerve fiber tangles (NFT) formed by aggregation of hyperphosphorylated tau proteins are two key pathological factors associated with AD (2). As the aging population increases, so will the number of AD patients in the world. However, there is no effective therapeutic option for AD.

The etiologies for most AD cases is unknown, except for 1–5% of cases where genetic differences have been identified. Several competing hypotheses have been proposed to explain the etiology of this disease. They include genetic, cholinergic hypothesis, amyloid hypothesis, and Tau hypothesis among others. In the last decade, more than 50 drug candidates have successfully passed phase II clinical trials, but none has passed phase III (3). The main challenge in the fight against Alzheimer’s disease is the absence of drugs that are capable of slowing disease progression. Since 2003, no new drugs have been approved for the treatment of Alzheimer’s disease. There are only limited studies of some dietary components improving AD conditions.

Long chain omega-3 polyunsaturated fatty acids (n-3 LC PUFAs) are long-chain fatty acids (20 or more carbons) whose first double bond is located at the methyl end after the third carbon. Docosahexaenoic acid (DHA), an omega-3 essential fatty acid, is a polyunsaturated fatty acid (C_22_H_32_O_2_) with six double bonds at positions 4, 7, 10, 13, 16, and 19. DHA and its derivatives have been shown to regulate gene expression levels of inflammatory mediators, as well as enzymes involved in lipid metabolism and Aβ processing. They are also important in preventing or retarding inflammatory aspects of AD pathology (4). Twenty-four week supplementation with 900 mg/day DHA was found to improve learning and memory functions in age-related cognitive decline. Therefore, it is a beneficial supplement for improving cognitive health with aging (5). Studies have reported potential therapeutic effects of DHA in preventing, regulating or improving AD progression.

Studies on the effects of DHA on AD pathology focused on behavioral aspects, which could not be associated with AD-related pathological indicators, and could not further explore the underlying mechanisms. Based on previous research findings, we used various comprehensive research methods to study the effects of DHA-rich mixed feeds on PS1/APP double transgenic AD mice models through behavioral experiments, *in vivo* NMR detection, liquid NMR detection and other methods. Learning and memory abilities, anxiety, Aβ formation and Tau hyperphosphorylation were evaluated.

The purpose of this study is to explore how DHA improves the symptoms of AD and try to discover the mechanism of treatment. The results of this study have certain guiding significance for the follow-up in-depth research on effective means of treating AD.

## Materials and Methods

### Animals and Treatment

Double transgenic AD mice models (2 × Tg-AD) carrying human gene mutants APPswePSEN1dE9/Nju and B6C3F1 WT mice were purchased from GemPharma Tech Co., Ltd. (Nanjing, China). Animals were raised in an animal facility under the conditions of 12-h light/12-h dark cycle at 22 ± 2°C with free access to food and water. The SPF grade standard feed and docosahexaenoic acid mixed feed were provided by CABIO Biotech (Wuhan) Co., Ltd. The 3-month-old 2 × Tg-AD male mice were randomly allocated into three groups: (i) AD control group; (ii) The 50 mg/kg DHA-treated AD group; (iii) The 300 mg/kg DHA-treated AD group, while age/gender-matched WT mice were allocated into two groups, which were the WT control and the 300 mg/kg DHA-treated WT groups. There were 12 mice in each group. The AD control and WT control mice groups were fed with the standard feed. For behavioral testing, all mice were fed to 7 months of age. Feeding was continued until 9 months of age (6 months of dosing), some mice were obtained for liquid nuclear magnetic resonance and functional magnetic resonance experiments while the remaining mice were euthanized and brain tissues as well as serum obtained for various biochemical and pathological experiments. Ethical approval was obtained from the Animal Ethical and Welfare Committee of Shenzhen University, China. All animal experiments were performed according to the animal experiment guidelines and regulations of Shenzhen University, China.

The low concentration of DHA is determined with reference to the new resource food 2010 No. 3 announcement issued by the China Food and Drug Administration, and the high concentration is determined with reference to the Chinese health food functional evaluation method (2020).

### Behavioral Tests

#### Morris Water Maze

The Morris water maze task was performed as previously described with some modifications (6) to assess spatial memory performance of the 9-month-old mice. The water maze was a blue tank (160 cm diameter, 50 cm height) filled with water (21–23°C) to a depth of 26 cm. At this temperature, mice are eager to find an opening through which they can escape from the water because they do not like swimming. The circular water pool was artificially divided into four equal areas, the first to fourth quadrant, and separated using an invisible platform (12 cm diameter, 1–2 cm below the water surface) in the middle of the third quadrant. During the five consecutive days of training, mice were gently released into the water in the first quadrant to start the test and were allowed up to 1 min to find the submerged platform. In case they could not mount the platform within 1 min, they would be guided to the platform for 10 s before being returned back to the cage. The escape latency and swimming speeds for the mice were recorded. After 24 and 72 h of training sessions, probe trials were performed in which the platform was removed. Each mouse was gently released into the first quadrant and searched freely for 2 m. Time that mice spent in the target quadrant (third quadrant) and the number of mice crossing the previous platform position were recorded. The trials were recorded using a video monitor connected to a computer. The test was run using a water maze software (Water Maze MT-200, Chengdu, China).

#### Open Field Test

The open field equipment was a 1.2 m × 1.2 m square PVC box, and the bottom of it was divided into 25 grids (5 × 5). The test was performed as previously described with some modifications (6). Briefly, a single mouse was placed in the middle grid of the box and timed for 3 mins. The number of grids that the mouse crossed and the number of rearing (the two front legs were raised at the same time) were recorded. To avoid affecting the experimental results, the smells were wiped off using ethanol before the next mouse experiment.

### Tissue and Serum Preparation

Mice were anesthetized using isoflurane and their brain tissues rapidly collected. The left hemisphere was fixed in 4% phosphate-buffered paraformaldehyde, while the right one was dissected into the hippocampus and cortex and stored at −80°C for the subsequent biochemical analyses. Blood samples were obtained by removing eyeballs of the mice. Blood was left to stand for 3–4 h and centrifuged at 15 × rpm for 30 min. Then, the supernatant was aspirated to obtain mice serum.

### Serum Biochemical Analysis

Biochemical analyses were performed using a fully automatic biochemical analyzer (iMagic-M7, ICUBIO). Test kits were manufactured by ICUBIO (Shenzhen, China), include Glutamate Pyruvate Transaminase (ALT) Assay Kit, Glutamate Oxaloacetate Transaminase (AST) Assay Kit, Low density lipoprotein cholesterol (LDL-C) Assay Kit, High density lipoprotein cholesterol (HDL-C) Assay Kit, Urea Assay Kit, Triglycerides (TG) Assay Kit. All procedures were performed according to the manufacturer’s instructions.

### Thioflavin T Staining

Brain sections were washed several times using ddH_2_O after which they were immersed in 0.001 g/mL ThT working solution (Sigma Aldrich, St. Louis, MO, United States) and stained at room temperature for 30 mins. Subsequently, sections were washed using ddH_2_O for 5 s and mounted with the anti-fluorescence quenching medium. Finally, sections were imaged using a confocal microscope (OLYMPUS FV1000).

### Silver Staining

After being washed several times using ddH_2_O, brain sections were treated with formic acid for 5 mins and washed three times again. Then, sections were placed in silver glycine solution (preheated at 37°C in advance) for 3–5 min, removed and the residual glycine silver solution on the sections quickly shaken off. Sections were put into reducing solution I (warmed to 45°C in advance), removed after a few seconds, quickly shaken, placed in reducing solution II for a few seconds (preheated to 45°C in advance) and cleaned using ddH_2_O. In case the staining background was too deep, it was treated using sodium thiosulfate solution and washed three times using ddH_2_O. The slides were mounted with anti-fluorescence quenching sealers and imaged by confocal microscopy (OLYMPUS FV1000, Olympus Corporation, Made in Japan).

### Statistical Analysis

Data were analyzed using GraphPad Prism software and presented as mean ± standard error of the mean (S.E.M.). Statistical significance were considered at *p < 0.05; ^**^p < 0.01; ^***^p < 0.001. Statistical analyses were performed by one-way ANOVA followed by the Student’s *t*-test.

## Results

### DHA Treatment Ameliorated Cognitive Impairment and Depression/Anxiety Behaviors in Alzheimer’s Disease Mice

**FIGURE 1:**
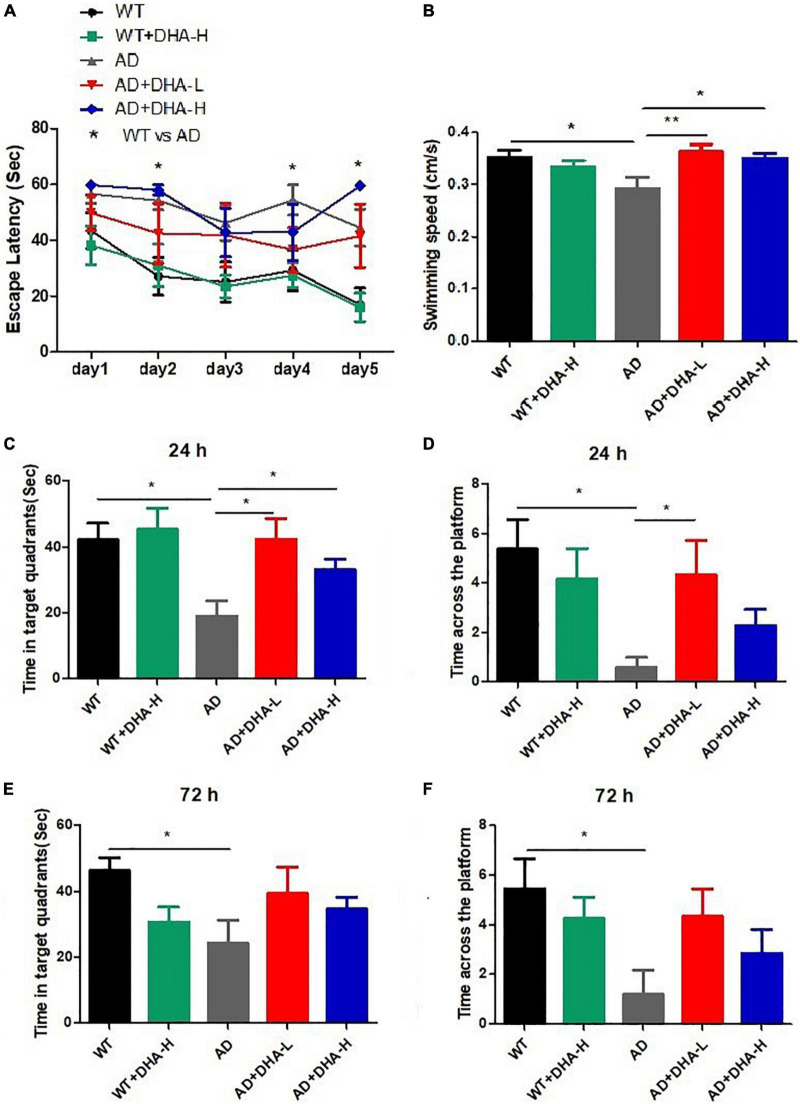
Effect of DHA treatment for 4 months on spatial memory and learning ability in 2 × Tg AD mice. **(A)** Escape latency; **(B)** average swimming speed; **(C)** time in target quadrant 24 h after 5-day training; **(D)** times crossing platform 24 h after 5-day training; **(E)** time in target quadrant 72 h after 5-day training; **(F)** times crossing platform 72 h after 5-day training (*p < 0.05; **p < 0.01). Student’s *t* test was used for statistical analysis. Error bars represent mean ± S.E.M. (*n* = 12).

The Morris water maze (MWM) was used to evaluate the effects of DHA treatment on spatial memory and learning abilities in 2 × Tg AD mice (6). After 4 months of DHA treatment, some mice groups exhibited progressively shorter escape latencies, while the WT mice treated with high DHA reached the shortest escape latency significantly on the fifth day (Figure 1A). Swimming speeds of mice in the WT, AD + DHA-L, and AD + DHA-L groups were significantly different from those of the AD group (Figure 1B), implying that DHA partially restored exercise and spatial learning abilities of AD mice. After 24 h of the detection period, target quadrant retention time of the WT, AD + DHA-L, and AD + DHA-L groups were significantly higher than those of the AD group, while there were no significant differences in the other groups (Figure 1C). Platform crossing numbers of DHA -treated mice group were not significantly higher than those of the other groups after 24 and 72 h. Similarly, DHA did not result in a significant increase in crossing numbers of mice in other groups (Figures 1D,F), implying that DHA could partially improve short-term memory of AD mice, but not their long-term memories. These findings show that feeding DHA for 4 months partially restored motor functions of AD mice and improved some of their learning and memory impairments.

**FIGURE 2:**
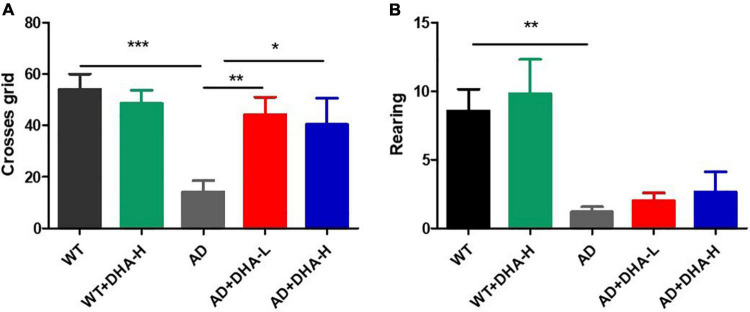
Effect of DHA treatment for 4 months on depression- and anxiety-related behaviors in 2 × Tg AD mice. **(A)** Times of crossing grids; **(B)** times of standing (*p < 0.05; **p < 0.01; ***p < 0.001). Student’s *t* test was used for statistical analysis. Error bars represent mean ± S.E.M. (*n* = 12).

The open field test (OFT) was performed to investigate the effects of DHA on depression- and anxiety-related behaviors of AD mice (6). After 4 months of DHA feeding, compared to WT mice, AD mice exhibited typical depression/anxiety behaviors, including significant reductions in the frequencies of grid crossing and rearing (Figures 2A,B). Compared to AD mice, the DHA treatment mice group exhibited significantly improved depression/anxiety behaviors, which was associated with a significant increase in grid crossing and feeding frequencies (Figures 2A,B). These behavioral MWM and OFT findings (Figures 1, 2) show that DHA treatment (including moderate or high DHA concentration) effectively improved some cognitive decline and depression/anxiety behaviors in AD mice.

### DHA Significantly Suppressed the Pathological Hallmarks of Alzheimer’s Disease Mice

**FIGURE 3:**
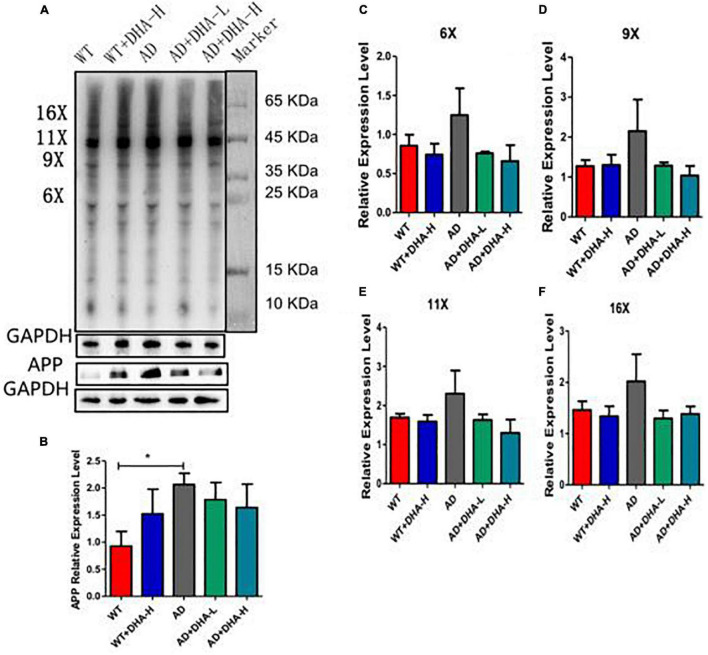
Detection of Aβ protein levels in mouse cortex tissue (*n* = 4). **(A)** Western blot detection of the expression levels of Aβ oligomers and APP in hippocampus; **(B)** Quantitative analysis of gray value of APP protein expression level; **(C)** Quantitative analysis of gray value of 6-fold oligomer of Aβ protein; **(D)** Aβ protein 9 Gray value quantitative analysis of fold oligomers; **(E)** Gray value quantitative analysis of Aβ protein 11-fold oligomers; **(F)** Gray value quantitative analysis of Aβ protein 16-fold oligomers (*p < 0.05).

Western blot was used to detect the expression levels of Aβ oligomers and APP protein in the hippocampus of mice. It can be seen from the results that the expression levels of Aβ protein and APP protein in AD group mice were significantly increased compared with WT group mice, and DHA administration for 4 months significantly decreased the AD + DHA-L and AD + DHA-H groups. The Aβ protein expression level and APP protein expression level of mice were not significantly different (Figure 3).

**FIGURE 4:**
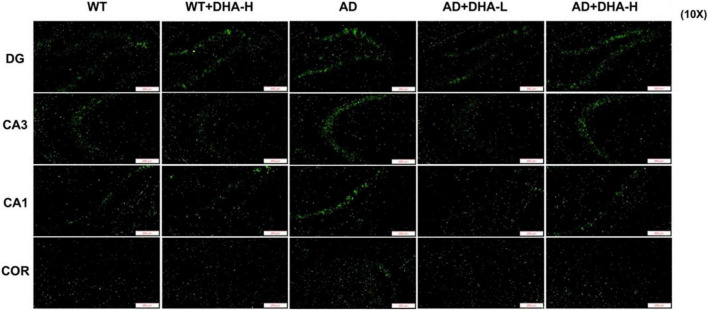
Effect of DHA treatment on deposition of Aβ in the hippocampus and cortex of 2 × Tg AD mice. CA3:CA3 area of mouse hippocampus; CA1, CA1 area of mouse hippocampus; Cor, cortical area; DG, dentate gyrus area of hippocampus (*n* = 4).

Interactions between Aβ and tau may be central to the development of Alzheimer’s disease (7) while APP/PS1 transgenic mice develop age-related accumulation of plaques and tangles in the brain (8). Detection of plaques in the hippocampus and cortex of mouse brain through thioflavin T staining, to some extent, reflects the accumulation and distribution of Aβ in the brain. From the findings, the areas occupied by Aβ plaques of WT mice were all reduced significantly in both the cortex and hippocampus, compared to AD mice. Compared to AD mice as control group, low or high DHA treatments reduced Aβ plaque aggregations in CA3, CA1, DG, and Cor of AD mice (Figure 4).

**FIGURE 5:**
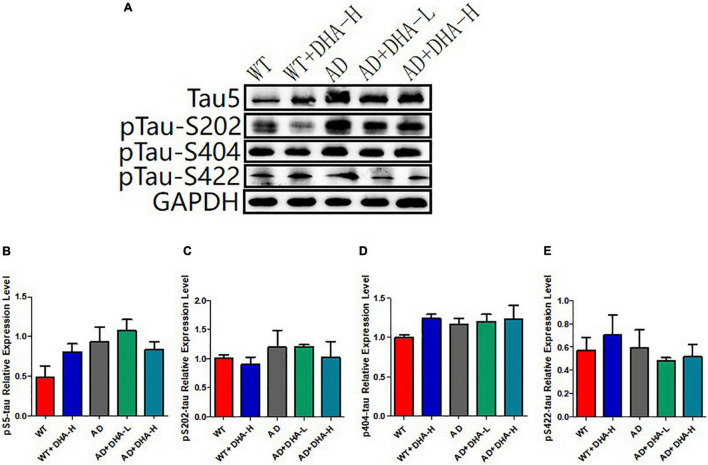
Detection of expression levels of total tau and phosphorylated tau protein in mouse hippocampus (*n* = 4). **(A)** Western blot detection of the expression levels of total tau and phosphorylated tau protein in the hippocampus of mouse brain; **(B)** Quantitative analysis of the gray value of total tau protein. **(C)** The expression level of phosphorylated protein at S202 of Tau. **(D)** Tau at the S202 site; the expression level of phosphorylated protein at S404; the expression level of phosphorylated protein at S422 of **(E)** Tau.

The results of Western blot showed that total Tau (Tau5) and Tau phosphorylation sites (S202, S404, and S422) had no significant changes in all groups of mice (Figure 5).

**FIGURE 6:**
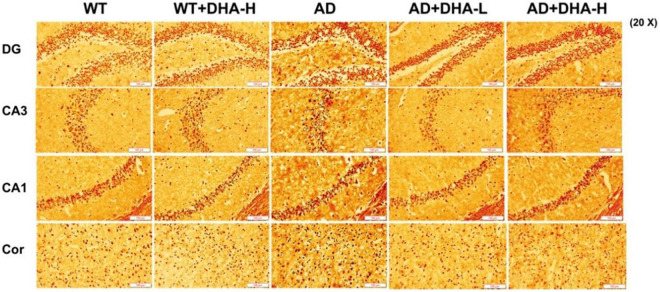
Effect of DHA treatment on neurofibrillary tangles in the hippocampus and cortex of 2 × Tg AD mice (*n* = 4).

Glycine silver staining was used to detect neurofibrillary tangles (NFTs) in the hippocampus and cortex of mouse brains. Formation of NFT was due to excessive phosphorylation of the tau protein, which caused it to entangle with each other after falling off the microtubule tangle of nerve fibers (9, 10). From the results, the NFTs of WT and DHA treated mice were significantly less than those of the AD group in CA3, CA1, DG, and Cor. However, DHA treated mice exhibited slightly worse outcomes than the WT group (Figure 6).

### DHA Had No Obvious Improvement Effect on Liver Functions, Kidney Functions, and Blood Lipid Levels in Experimental Mice

Apolipoprotein A1 (ApoA1) and apolipoprotein B (ApoB) are lipid-related indicators (11, 12). ApoA1 is a high-density lipoprotein whose main role is to transport lipids from tissues and organs to the liver, and the liver decomposes the lipids (13). ApoA1 is negatively correlated with atherosclerotic cardiovascular disease. ApoB exists on the surface of low-density lipoprotein and is used to detect and take up low-density lipoproteins, increasing coronary atherosclerotic heart disease incidences even at low levels (12). Triglyceride (TG), also known as neutral fat, is the main source of energy in the body. Its content increases with age, especially for middle-aged and elderly people whose weight exceeds the standard. High triglyceride levels can easily result in thick blood and thrombosis leading to high blood pressure, gallstones, pancreatitis, and Alzheimer’s. Apolipoprotein A1 (ApoA1) is mainly present in form of HDL and is the main apolipoprotein of HDL. It is mainly synthesized in the liver and small intestines. Under normal circumstances, high levels of HDL are better than low levels, which helps prevent hyperlipidemia, fat deposition and arterial Atherosclerosis. As the main apolipoprotein of LDL, Apolipoprotein B (ApoB) has various physiological functions, including the synthesis, assembly and secretion of VLDL rich in triglycerides. Clinically prescribed ApoB refers to ApoB100, which is better at low levels under normal conditions. We found that DHA had no significant effects on the levels of triglycerides, HDL, and LDL in each group of mice.

**FIGURE 7:**
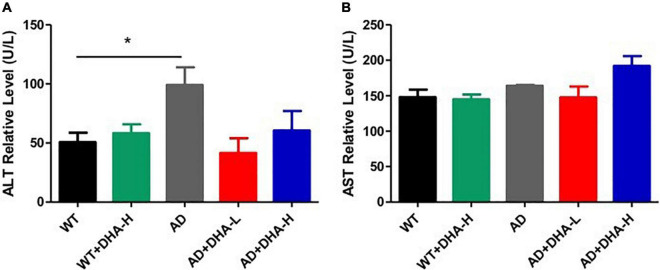
Effects of DHA treatment on liver - indicators in 2 × Tg AD mice. **(A)** Alanine aminotransferase; **(B)** aspartate transferase (*p < 0.05). The student’s *t* test was used for statistical analyses. Error bars represent mean ± S.E.M. (*n* = 6).

Aspartate transferase (AST) and alanine aminotransferase (ALT) are used to evaluate liver functions (14). Alanine aminotransferase (ALT) is an enzyme involved in the metabolism of human proteins. When liver cells are damaged, ALT is released from cells. We found that liver functions of AD mice in the WT group were significantly impaired. Cell damage can cause serum ALT activity to double, therefore, determination of ALT activity is one of the most sensitive indicators of liver cell damage. After adding DHA, serum ALT activities of AD + DHA-L and AD + DHA-H groups were significantly suppressed, which was basically the same as that of the WT group. Serum ALT activities of the WT + DHA-H and WT mice groups were significantly altered. Therefore, high-dose DHA had no toxic effects on WT mice, while low-dose DHA had a certain protective effect on the livers of AD mice (Figure 5A). Aspartate aminotransferase (AST) was formerly known as glutamic oxaloacetic transaminase (GOT). AST has two isoenzymes, ASTs and ASTm, which exist in soluble cytoplasm and mitochondria, respectively. When liver cells are mildly damaged, ASTs increase significantly, and in severe injuries ASTm appear in the serum in large quantities. We found that there were no significant differences in AST levels among the WT, AD, and DHA treatment groups, implying that drug treatment had no toxic and side effects on mice livers (Figures 7A,B).

**FIGURE 8:**
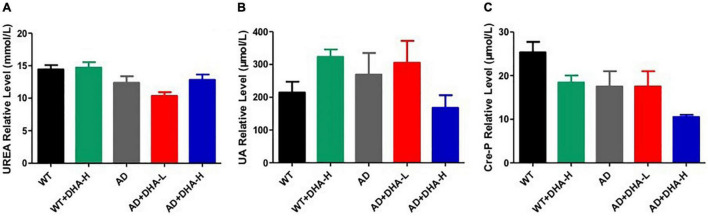
Effect of DHA treatment on kidney- indicators in 2 × Tg AD mice. **(A)** Urea; **(B)** auric acid; **(C)** Cre-P. Student’s *t* test was used for statistical analyses. Error bars represent mean ± S.E.M. (*n* = 6).

For kidney functions, uric acid (UA) and urea are very important. UA is the final product of purine metabolism. Alterations in UA levels can fully reflect the body’s metabolic, immune and other functions (15). Urea is a product of human protein catabolism (16). Excessive uric acid and urea levels indicate liver and kidney damage, while an increase in UA levels but within the normal range can hinder the onset of AD (17). In this study, there were no significant differences in UA and urea levels among the WT, AD, and DHA groups, implying that drug treatment had no toxic or protective effects on kidney functions (Figure 8).

From the analysis of serum biochemical indicators, DHA did not improve liver as well as kidney functions and neither did it affect blood lipid levels of experimental mice. In addition, it had no toxic effects on experimental mice.

### DHA Had Little Effects on Inositol, Glycine, and Taurine

Inositol, glycine, and taurine are common small molecules in the brain. They are associated with brain development, neurotransmission among others (18–20). The results of the concentration of Myo-instal in different brain regions showed that the WT group did not change significantly compared with the WT + DHA-H group, and the concentration of Myo-instal in the WT group showed an upward trend compared with the AD group. For mice in the AD + DHA-L, AD + DHA-H, and AD groups, they were concentration of Myo-instal down-regulated in the striatum, cortex and epithalamus, with significant differences. Since the specific role of concentration of Myo-instal is not yet clear, the result needs further verification.

Concentrations of glycine in different brain regions and the ratio of WT group to WT + DHA-H were all down-regulated. Moreover, the concentration of glycine in AD mice were also down-regulated. There was almost no change in glycine levels in the AD + DHA-L and AD groups, but the content of AD + DHA-H group has changed. These findings indicate that high-dose DHA may not have much effect on normal mice, but it can improve glycine levels in the brains of AD mice.

**FIGURE 9:**
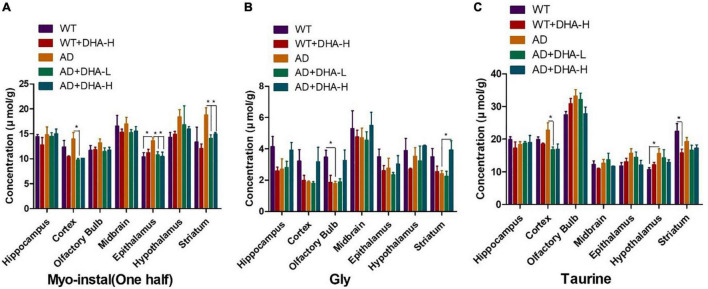
Effects of DHA treatment on common small molecules in the brain in 2 × Tg AD mice. **(A)** Myo-Inositol, **(B)** glycine, **(C)** taurine. Student’s *t* test was used for statistical analyses. Error bars represent mean ± S.E.M. (*n* = 4). *P < 0.05.

**FIGURE 10:**
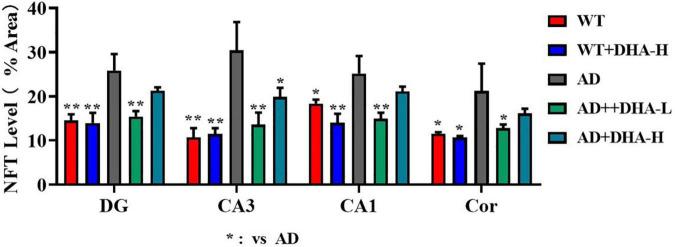
Effects of DHA treatment on NFT level in the brain in 2 × Tg AD mice. *p < 0.05, **p < 0.01. Error bars represent mean ± S.E.M. (*n* = 6).

**FIGURE 11:**
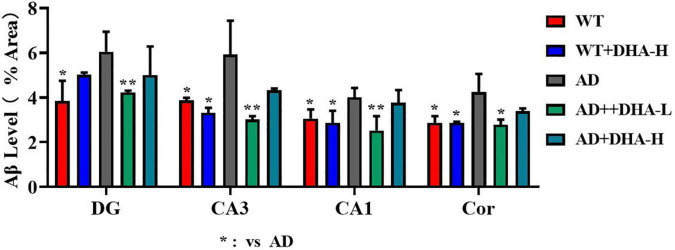
Effects of DHA treatment on Aβ level in the brain in 2 × Tg AD mice. *p < 0.05, **p < 0.01. Error bars represent mean ± S.E.M. (*n* = 6).

Compared to the WT group, the WT + DHA-H group exhibited significantly decreased concentrations of taurine in the striatum. The concentration of taurine results in other brain regions were different from the wild type group. WT group compared to the AD group, taurine in the HYP area was significantly up-regulated. After feeding with DHA, mice in the AD + DHA-L group exhibited significantly down-regulated taurine levels in the cortical area, while significant effects were not observed in mice in the AD + DHA-H group (Figure 9). Detection of neurofibrillary tangles (NFTs) in the hippocampus and cortex of mouse brain by silver glycinate staining. The NFT positive areas were evaluated using Image-Pro Plus Software. Western blot detection of Aβ protein expression in hippocampus of mouse brain. The pictures represent NFT level (Figure 10) and Aβ level (Figure 11) in the mice brain.

## Discussion

Alzheimer’s disease is a progressive neurodegenerative disorder that is commonly associated with the onset of dementia in elderly people. The main clinical symptoms for AD include progressive memory loss and cognitive impairments (21). Typical pathological features include accumulation of Aβ plaques in brain tissues, nerve fiber tangles in brain cells, and neuronal loss among others, resulting in inflammation, oxidative stress, impairments in vasorelaxation, and progressive brain atrophy (1, 7, 22, 23). DHA has been shown to enhance brain development, cognition, and learning behaviors (24). After DHA digestion, it can bypass tight junctions of the blood brain barrier (BBB) and enter the brain through inner membrane lobules. Therefore, improving Alzheimer’s disease outcomes through dietary intake of DHA is safe and effective. Free DHA in dietary is transported across the outer membrane leaflet of the blood-brain barrier (BBB) *via* passive diffusion, attenuates the brain inflammatory response that leads to AD. A limited number of studies have evaluated the mechanisms through which DHA interferes with Alzheimer’s disease progression. In this study, we used APP/PS1 transgenic mice model that developed age-related accumulation of plaques and tangles in the brain, showing some relatable symptoms of Alzheimer’s disease to test the hypothesis that DHA is effective in treating Alzheimer’s disease. Compared to AD control mice group, DHA improved spatial learning, memory ability and anxiety of mice. It also suppressed the accumulation of some Aβ oligomers and the formation of neurofibrillary tangles. In mice models, DHA did not interfere with liver and kidney functions, and it improved AD symptoms by improving blood lipid functions and preventing cardiovascular and cerebrovascular diseases.

Not only is DHA an essential factor for the healthy growth and development of infants and young children, it is also involved in improving cardiovascular health, reducing the symptoms associated with rheumatoid arthritis and depression, and improving human cognition. Large amounts of DHA are contained in the cerebral cortex and retina, especially in the gray matter and photoreceptor cells of the cerebral cortex. These DHAs are mainly in the form of phospholipids (25). DHA is very important in brain development (26). With cell membrane formation, neurons continue to form axons and dendrites. The growing cell membrane must be relatively fluid, and DHA is very fluid. Moreover, synapses of the most important functional units in the brain circuit are preferentially composed of DHA-rich cell membranes (27). DHA is an essential factor for the differentiation and migration of neurons as well as glial cells during brain development. It is also important in stimulating myelination and synapse. In addition, DHA is involved in neural cell differentiation (28), neural development (29) and in preventing synapse loss (30). Alzheimer’s disease is prevalent in Western countries. Due to the association between DHA and brain development, and the relatively low intake of omega-3 fatty acids in these countries, there is a need to determine whether increasing DHA intake can reduce AD risks and improve cognitive repair in patients with mild AD. After death, in death patients, DHA levels decrease in specific areas of the brain, such as the pons, white matter, and especially in the frontal gray matter and hippocampus (31). In addition, since DHA contains six double bonds, it is very sensitive to lipid peroxidation. Elevations in peroxidation products of DHA in the brains of AD patients assumes that the loss of DHA can be attributed to the increase in oxidative stress (32). This lipid change occurs in the latter stages of AD, but also in the early stages of the disease (33). PUFA plays important roles in the prevention of AD, other types of dementia, and cognitive impairment (34). The prevention of AD by DHA is mostly based on the amyloid hypothesis (3, 35). Dietary DHA was shown to be involved in improving learning-related memory deficiencies in Aβ peptide-infused AD rat models. DHA pre-administration ameliorated the deterioration of learning ability-related memory in AD rat models (36). Moreover, dietary DHA is protective against Aβ production, accumulation, and its potential downstream toxic effects (37). Cell cultivation experiments revealed that DHA down regulates the secretion of Aβ peptides in aging human neural cells (38). Dietary DHA was associated with significantly increased levels of memory in the hippocampus and frontal cortex, which was associated with the protection of synaptic proteins. Both parameters were strongly correlated with learning as well as memory capacities and were implicated in the prevention of Aβ oligomer-induced impairments. Therefore, DHA is important in AD prevention (39). DHA was shown to protect against and ameliorate learning ability impairments in Aβ1–40 infused AD rat models, with concurrent increases in DHA levels and decreases in lipid peroxide levels and reactive oxygen species in the cortico-hippocampal tissues (40).

The mechanisms through which DHA affects AD symptoms may involve indirectly influencing blood vessels to prevent Aβ production. A diet rich in DHA altered the behaviors and cognitive abilities, cerebral hemodynamics [relative cerebral blood volume (rCBV)] as well as Aβ deposition levels in APPswe/PS1dE9 mice at 8 and 15 months of age (41). The study found that DHA altered rCBV in 8-month-old APP/PS1 and wild-type mice [AU1]. In 15-month-old APP/PS1 mice, DHA supplementation improved spatial memory, reduced Aβ deposition, and slightly increased rCBV, implying that a diet rich in DHA can reduce the pathological symptoms associated with AD. Therefore, long-term consumption of DHA-rich foods can improve AD symptoms. The Framingham Heart study showed that high plasma DHA levels can reduce the risk of AD development (42).

Moreover, adequate DHA intakes can reduce the risk of AD development (43–45) while dietary intake of n-3 PUFA may suppress cognitive declines (46, 47). Freund-Levi (48) performed a trial and proved the positive effects of DHA supplementation on cognition of very mild AD patients. In APP transgenic mice, brain Aβ levels were found to be decreased after dietary DHA supplementation (37, 49). It has been postulated that DHA supplementation increases the amounts of neuron sorting protein LR11, which regulates the processing of APP and the subsequent reduction in Aβ production (50). In addition, DHA supplementation in tg2576 AD mice depleted of DHA improved memory acquisition (30). The study by Hooijmans corroborates these findings (51). In their study, they used open-air experiments to determine exploratory behaviors and used the Morris water maze (MWM) and 8- and 15-month-old wild-type and double-transgenic APPswe/PS1dE9 mice. The 12 circular orifice plates were used for spatial learning and memory assessments (41).

The water maze experiment is the most classic experiment to test spatial learning and memory abilities of mice (51). The escape latency of the 5 days’ spatial training, swimming speed, time in target quadrant and the number of crossing platform reflects learning abilities, movement abilities, and spatio-memory abilities (52). In this study, the escape latency for mice in each group was shortened day by day. However, some mice in the same cage were injured in a fight, mice in the AD + DHA-H group took more time to find the platform on the 5th day than in the previous 4 days due to injuries in some mice. At 24 and 72 h after training, the time spent in the platform quadrant of the mice in each DHA treatment group was significantly increased compared to WT and AD mice. Compared to 72 h, residence time of mice in the 24 h treatment group and the AD group was significantly increased. The WT + DHA-H group exhibited an increasing trend compared to the WT group, however, the difference was not significant. The number of times mice in each treatment group crossed the platform was also better at 24 h than at 72 h. Compared to the AD group, the number of times mice in the WT group crossed the platform was significantly high. Finally, 24 and 72 h after training, the number of times mice in each treatment group crossed the platform was also better at 24 h than at 72 h. Results for mice in the WT group were more significant than those in the AD group. Compared to AD group mice, the number of times that mice in the AD + DHA-L group crossed the platform was significantly increased. In this study, low-dose DHA improved memory and learning abilities of AD mice, without significant changes in other groups.

The open field experiment, which exploits mice innate avoidance to open fields and exploration of new things, is used to evaluate spontaneous activities and anxiety in mice (6). In this study, we found that long-term DHA supplementation increased the number of rearing and crossing grids of AD mice, implying that the spontaneous activity and anxiety of AD mice were improved by DHA treatment. Combining the results from the water maze experiment and the open field experiment, long-term DHA supplementation had a certain therapeutic effect on impaired cognitive and motor abilities of AD mice.

As earlier mentioned, Aβ and hyperphosphorylated tau are two major toxic proteins of Alzheimer’s disease (7). Aβ is attributed to continuous shearing of APP proteins by β-amylase and γ-secretase. Large amounts of Aβ precipitate outside neurons to form senile plaques (SP) (7, 22, 53). Tau promotes the assembly of microtubules and maintains cytoskeleton functions. Over-phosphorylated tau not only loses its biological functions but also dissociates from the microtubules and aggregates with each other, eventually forming a large number of NFTs (9, 54). Aβ has strong toxic effects and induces tau hyperphosphorylation, which leads to neuronal degeneration, dysfunction, and death (55, 56). In this study, through brain section thioflavin and silver staining, we found that the number or area of beta amyloid in DHA-fed AD mice was reduced in certain brain regions, showing the Aβ clearance effect of DHA. The number of NFTs in AD mice was also decreased with increasing DHA concentrations. Neuronal damage and death, especially in brain learning and memory-related areas, are pathological features of AD (1). Therefore, DHA suppressed neuronal damage by reducing Aβ and inhibiting tau protein hyperphosphorylation, which improved cognitive abilities and mobility of AD mice.

Obesity, hypertension, cardiovascular and cerebrovascular diseases increase the risk of AD (57–59). Lipoproteins are complexes of different lipids and proteins that act on the transport and clearance of lipids or lipid-related molecules from circulation (60). Lipoproteins also play a vital role in brain functions. High-density lipoproteins (HDL) and their main protein component, ApoA1, are directly involved in the outflow of cholesterol from the brain (12, 61). Overexpression of human ApoA1 in circulation was shown to prevent learning and memory impairments in APP/PS1 transgenic mice, partly by suppressing neuroinflammation and cerebral amyloid angiopathy (12). In addition, ApoB was found to be elevated in AD plasma and serum, and over expression of ApoB in transgenic mice triggered apoptosis and neurodegeneration in the brain (62–64). In this study, there were no significant differences in ApoA1 and ApoB levels between the WT and AD groups. Moreover, after DHA supplementation, differences in ApoA1 and ApoB levels were not significant, implying that DHA had no effect on the apolipoproteins.

Analysis of serum biochemical indicators showed that DHA had no effects on blood lipid levels, kidney functions and liver functions of experimental mice. AST and ALT are the most sensitive indicators of liver cell damage. When liver cells are severely damaged, AST and ALT levels are significantly elevated in serum (14). In this study, neither DHA supplemented AD mice nor WT mice had significantly elevated AST and ALT levels, and there were no differences between the groups, implying that DHA did not impair liver functions.

Among the possible markers for age-related cognitive decline, UA is controversial because it has antioxidant properties. However, elevated UA levels increases the risk of gout and cardiovascular disease and affects kidney functions. Serum UA levels in AD patients were found to be significantly low than in healthy controls (14, 65). In this study, there were no significant differences in renal function indices in WT, AD and DHA treatment groups, implying that DHA had no toxic effects on kidney functions, and no obvious protective effects.

Inositol is associated with signal transduction, and its imbalance leads to neurological disorders (18). Inositol was shown to improve pathological characteristics of Alzheimer’s disease animal models and reversed cognitive impairments (66, 67). However, we found that inositol was stably expressed in different groups. Glycine is a neurotransmitter with anti-inflammatory, signaling, and cellular immunity functions (19). Inhibition of glycine transporter-1 (GlyT1) elevated glycine levels, thereby inhibiting cognitive impairments in Alzheimer’s disease animal models (68). We also found that high-dose DHA may not have a significant effect in normal mice, but it improved glycine levels in the brains of AD mice, which may be a good phenomenon. Due to aging, taurine levels in mammalian brain decrease, affecting memory ability (69).

In this study, taurine levels in the brains of AD mice fed with DHA did not significantly improve.

## Conclusion

(1) DHA suppressed neuronal damage by reducing Aβ and inhibiting tau protein hyperphosphorylation, which improved cognitive abilities and mobility of AD mice.
(2) DHA has a certain effect on lowering blood lipid levels and may have a positive effect on the prevention of vascular diseases, which may improve Alzheimer’s disease outcomes.

## Data Availability Statement

The original contributions presented in the study are included in the article/supplementary material, further inquiries can be directed to the corresponding author.

## Ethics Statement

The animal study was reviewed and approved by Animal Ethical and Welfare Committee of Shenzhen University.

## Author Contributions

MX: conceptualization, methodology, project administration, and writing – review and editing. WX: data curation, writing – original draft preparation, and formal analysis. YC: writing – review and editing and investigation. NP, SL, and YZ: writing – review and editing. XD: resources, data curation, and formal analysis. BL: project administration. YH and XL: supervision. All authors contributed to the article and approved the submitted version.

## Conflict of Interest

MX, WX, SL, YZ, BL, and XL were employed by CABIO Biotech (Wuhan) Co., Ltd. The remaining authors declare that the research was conducted in the absence of any commercial or financial relationships that could be construed as a potential conflict of interest.

## Publisher’s Note

All claims expressed in this article are solely those of the authors and do not necessarily represent those of their affiliated organizations, or those of the publisher, the editors and the reviewers. Any product that may be evaluated in this article, or claim that may be made by its manufacturer, is not guaranteed or endorsed by the publisher.
